# Supplementary material for: Honokiol and Nicotinamide Adenine Dinucleotide Improve Exercise Endurance in Pulmonary Hypertensive Rats Through Increasing SIRT3 Function in Skeletal Muscle
Source: Int J Mol Sci. 2024 Oct 29;25(21):11600. doi: 10.3390/ijms252111600 (PMC11545838; doi:10.3390/ijms252111600)
Supplement: Supplementary file 1 [file ijms-25-11600-s001.zip › ijms-3257549-supplementary.docx]

Supplementary Materials

Honokiol and Nicotinamide Adenine Dinucleotide Improve Exercise Endurance in Pulmonary Hypertensive Rats Through Increasing SIRT3 Function in Skeletal Muscle

Min Li ^1,^ *, B. Alexandre McKeon ^1^, Sue Gu ^1^, Ram Raj Prasad ^1^, Hui Zhang^1^, Sushil Kumar ^1^, Suzette Riddle ^1^, David C. Irwin ^1^ and Kurt R Stenmark ^1,^ *


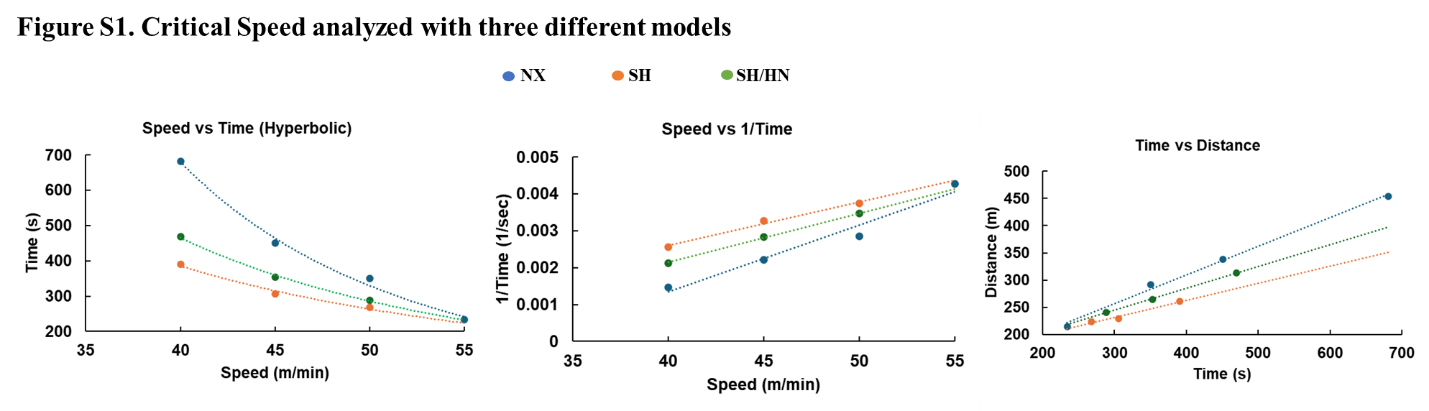


Figure S1. The representative critical speed of each group (NX, SH and SH/HN) analyzed with three different models: 1) the hyperbolic speed model [Speed= (D´⁄time) + CS] where the asymptote of the hyperbolic curve is the CS and the curvature constant is the D´ (finite distance), 2) the linear 1/time model (Speed = D´ x 1⁄time + CS) whereby the treadmill speed used for the constant speed test is plotted as a function of the inverse of time to fatigue, and the y-intercept of the regression line yields the CS, and the slope is the D´, and 3) the linear distance/time model, where the distance run by the mouse is plotted against the time to fatigue (Distance = CS x time + D´) and the slope of the regression line is the CS and intercept is the D´.

| **Table S1**. Rat primer sequences for real-time PCR | | |
| --- | --- | --- |
| Genes | Forward (5’-3’) | Reverse (5’-3’) |
| Glut1 | TGGCCAAGGACACACGAATACTGA | TGGAAGAGACAGGAATGGGCGAAT |
| Ldha | GGCATGGCTTGTGCCATCA | AGGCTGCCATGCTGAAGATC |
| Sdf1 | TGTGCATTGACCCGAAATTA | TCCTCAGGGGTCTACTGGAA |
| Vcam1 | TGCACGGTCCCTAATGTGTA | TGCCAATTTCCTCCCTTAAA |
| Il6 | AGTTGCCTTCTTGGGACTGA | CTGGTCTGTTGTGGGTGGTA |
| Mcp1 | CTGTAGCATCCACGTGCTGT | CCGACTCATTGGGATCATCT |
| Mki67 | GGATGTGAGAGAAGAGCCTTCG | TGCCTGTCACTCTGCCTGATG |
| Cdk1 | GGGAACAGAGAGGGTCCGTT | ATCTGGCCAGTAGTCCTGTG |
| Tnc | GCATCGGTCACTGGATACCT | CAGGCTGTAGGAGGTGGTGT |
| Fn | CAAGGTCCGAGAAGAGGTTG | CCGTGTAAGGGTCAAAGCAT |
| Nppa | TGCAACAGCTTCCGGTACCG | TGTGACACACCGCAAGGGCT |
| Nppb | GGTCTCAAGACAGCGCCTTC | ACAACCTCAGCCCGTCACAG |
| Acta1 | TCACTTCCTACCCTCGGCAC | AGGCCAGAGCCGTTGTCACA |
| Murf1 | ACAACCTCTGCCGGAAGTGT | CCGCGGTTGGTCCAGTAG |
| Atrogin-1 | CATCCTTATGCACGCTGGTC | GGTCTCCATTCGATACACCCA |
| Cathepsin-L | GAC GGT GGG GCC TAT TTC TG | TTC CGG TCT TTG GCT ATT TTG A |
| Foxo3a | TGAGGAAAGGGGAAATGGGC | TGGGTTAGGAAGATGGCGTG |
| Foxo1 | ATTCGCCACAATCTGTCCCT | TTTCTTAGCAGCCCGTCCTC |
| Hk2 | GCCTGGTTTCAAAGCGGTCG | TCCGTGAATAAGCAGGCGAT |
| Hprt | AAGCTTGCTGGTGAAAAGGA | CAAGGGCATATCCAACAACA |
